# Evolution of Rate of Force Development (RFD) in the Isometric Deadlift Exercise Among Primary and Secondary Education Students

**DOI:** 10.3390/sports14020060

**Published:** 2026-02-04

**Authors:** Julio Martín-Ruiz, Ignacio Tamarit-Grancha, Amparo Aguilar-Prima, Laura Ruiz-Sanchis

**Affiliations:** 1 Department of Health and Functional Assessment, Catholic University of Valencia, 46900 Valencia, Spain; 2 Department of Physical Preparation and Conditioning, Catholic University of Valencia, 46900 Valencia, Spain; ignacio.tamarit@ucv.es; 3 GIEPAFS Research Group, Catholic University of Valencia, 46900 Valencia, Spain; amparo.aguilar@mail.ucv.es; 4 Department of Sports Management and Physical Activity, Catholic University of Valencia, 46900 Valencia, Spain

**Keywords:** rate force development, deadlift, Physical Education

## Abstract

Strength is a central axis of physical activity and sets the right evolutionary direction for children and adolescents, creating adaptations that determine functional health in adulthood. Therefore, its development and supervision are essential in the future. This study aimed to measure the rate of force development (RFD) in a sample of primary and secondary school children using the deadlift exercise. In a mixed sample of 227 students aged 9–16 years, two attempts of the isometric deadlift exercise were performed using a hand-held dynamometer. Pain perception was recorded after each attempt was made. RFD evolved in both stages, with a greater difference in boys in Secondary School (*p* = 0.0017), and no additional differences in the rest of the variables between sexes and stages. Pain during execution was infrequent in this sample and showed no consistent association with RFD or maximal force in adjusted exploratory models. Key outcomes (maximal force and RFD_{0–200}) were summarized by sex and educational stage; RFD was computed as ΔF/Δt over 0–200 ms from contraction onset. The main findings highlight the expected progression of strength, especially in boys, and support the deadlift as an accessible alternative for strength assessment in educational settings and health-oriented recreational activities, given its low incidence of pain during execution in this population.

## 1. Introduction

Strength is a fundamental component of physical fitness [1], and muscle activity is the primary variable of physical fitness [2].

Their way of working has undergone a transformation over the past decades, shifting from methodologies with lighter workloads and moderate mobilization rhythms [3] to being prescribed at any age with great intentional speed and high-impact loads [4], improving contractile capacity and preventing sarcopenia in adults.

Maximal force and rate of force development (RFD) are commonly used to characterize strength capacity and explosive strength, respectively, with RFD reflecting the speed at which force can be produced in the early phase of contraction [5,6].

When this phenomenon occurs, it can be said that there is proper intramuscular coordination, which is evidenced intermuscularly in a technical movement that is smoother and able to project all the strength potential that the individual possesses [7,8].

This is important not only in sports activities that depend on exceptionally short activation times, but also in recreational practice aimed at health [9], in which achieving the highest individual psychophysical condition and focusing on preventive aspects are among the priorities. An example of this can be seen in published studies in various fields showing that greater explosiveness helps with faster recovery in cases of muscle atrophy [10] and neurodegenerative diseases [11], that there is a correlation between lower pain during an activity and shorter contraction time [12], and that good conditioning improves response times in dynamic destabilization actions [13].

When it comes to assessment, there are some key aspects to keep in mind, with the most important being the impulse in Newtons/sec or the best RFD in 50 ms, which provides an idea of initial explosiveness [14], and they are better at higher values and without fluctuations [15].

The variation among these details reveals the subject’s profile when displaying voluntary muscle strength. Low initial impulse values, or a wide gap between the onset and end of the signal, indicate the need to employ rapid force methods [16], in which the output per unit of time is prioritized. Along these lines, they suggested that there is room for improvement in intra- and intermuscular factors until reaching the optimal motor unit (MU) recruitment value of the ideal 80%, compared to the initial 30–35% of a healthy, untrained individual [17].

The type of contraction affects the test outcome. Dynamic contractions have a strong correlation with force production and can be performed with dynamic segmental exercises [18] and open kinetic chains using tools such as inertial devices that allow monitoring of the initial force and its development throughout the entire movement [19,20].

In the case of well-studied isometrics [21], their relationship is one of maximum correlation with a very low margin of error [22]. In environments with limited experience, the selection of multi-joint exercises is a more common option because of better technical assimilation and comparison with Olympic exercises, which are the most commonly used [23,24].

The squat and deadlift exercises meet these characteristics. Squats develop overall muscle mass, require a high degree of proprioception [25], and are easy to fall back into technical errors that would alter the action of the agonists (e.g., lordosis and reduced mobility of the triceps surae). As an alternative to this exercise, the deadlift helps to reduce these limitations and has some qualities: it is simple to teach, easy to reproduce, and compatible with the available equipment. This variable is especially important at early ages, as the paravertebral muscles represent the main agonist group along with the lumbar multifidus [26] and present other synergies that make it a global endeavor [27,28].

When searching for background information on the calculation of RFD in the deadlift only in adults [29,30] but not in school-aged individuals, the aim of the present study was to calculate the RFD in the isometric deadlift exercise in a mixed sample of students aged 9–16 years.

The hypothesis of this study is based on the idea that older age will be associated with a higher RFD and lower variability, with differences between sexes.

Its calculation could help identify the quality of force application in the technical gesture and detect areas for improvement related to the rapid application of force that could be addressed in PE classes, implicitly developing preventive muscle strength. Therefore, this study aimed to describe the force–time characteristics during an isometric deadlift task in school-aged participants and examine the differences by sex and educational stage.

## 2. Materials and Methods

### 2.1. Problem Statement

To observe the strength development profile in a sample of Primary and Secondary Education students using the deadlift exercise, an analytical, comparative, and cross-sectional study was designed. Authorizations were collected from the schools and students’ parents because the students were minors.

The measurement sessions were conducted during Physical Education (PE) class hours. The principles of the Declaration of Helsinki were followed, and the study was approved by the Ethics Committee of the Catholic University of Valencia (approval code: UCV/2024-25/116).

### 2.2. Participants

The sample consisted of 227 students from Primary Education (96), 47 boys and 49 girls, and Secondary Education (131), 80 boys and 51 girls, from educational centers in Valencia, Spain. The inclusion criteria were as follows: age between 9 and 16 years, regular attendance in PE classes, and no engagement in physical activity on the day of the test or the day prior.

The exclusion criteria were as follows: injury at the time of the study, convalescence for less than a month before the test, or exemption from participating in PE for medical reasons.

### 2.3. Procedure

After obtaining informed consent, the measurements were conducted in a single session during PE class.

At the beginning, anthropometric measurements were taken by the principal investigator (ISAK-I) for wingspan, biacromial distance, weight, height, and leg length. Wingspan was calculated using a measuring tape fixed to the wall, measuring the distance between both middle fingers with shoulders abducted at 90° and elbows extended. For the biacromial distance, the participants were asked to remain still in the anatomical position, and the distance between both acromions was measured.

Height was measured using a stadiometer (Seca 213; Hamburg, Germany) with the chin positioned horizontally at the Frankfort plane. For weight measurement, a digital scale (Seca 213, Hamburg, Germany) was used, and the result was obtained after two seconds of static position. Finally, leg length was measured using an anthropometric tape (Lufkin, Apex Tool Group, W606PM, Querétaro, Mexico) with the participant in a seated position and the knee extended while the foot was supported in plantar flexion. The distance was measured from the greater trochanter to the toes.

After this phase, the deadlift technique was explained to the students in detail, with particular emphasis on body positioning and key elements to consider when generating force. Next, a standardized warm-up was conducted, consisting of exercises aimed at reducing spinal viscosity and increasing muscular compactness: Cat-camel (6 reps), Bird-dog (6 reps), and front plank (15 s). Subsequently, global joint mobility exercises were performed, followed by general muscle activation exercises transferable to the deadlift technique and adapted for beginners: half squat without weight (10 reps), shoulder flexion-extension (10 reps), and 3 kg medicine ball throws with extension of knees, hips, and paravertebral muscles (6 reps). Subsequently, the deadlift movement was measured using the sensor.

### 2.4. Deadlift

An isometric deadlift test was performed using a validated [31] non-elastic dynamometer (Force Sensor Kit; ChronoJump, Barcelona, Spain). The participants stood in a bipodal stance on a steel plate marked in centimeters, with their feet at the biacromial width.

**Figure 1:**
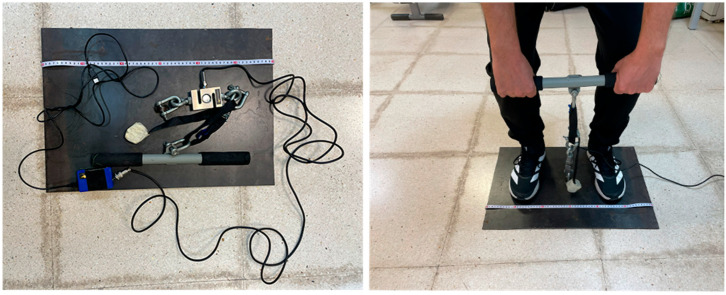
(**Left**): Steel plate, dynamometer, strap, and grip bars. (**Right**): Foot position and hand grip.

They were required to hold a rigid weightlifting bar attached to a strap to adjust the distance, which was connected to a dynamometer and the latter to a steel plate (Figure 1).

The isometric deadlift was chosen because it is a globally oriented, technically accessible pulling pattern that can be standardized and safely administered in school settings, while enabling the collection of force–time variables (e.g., maximal force and RFD) with minimal logistical requirements [32].

Participants performed the deadlift with approximately 90° flexion, the position of maximum force application for global lower limb exercises [33,34], pushed their hips back, and kept their spine extended paravertebrally with an upward gaze to avoid lumbar inversion and ensure that their effort targeted the appropriate muscles. To minimize pretension and countermovement artifacts, the strap length was adjusted so that the initial tension did not change the participant’s posture.

**Figure 2:**
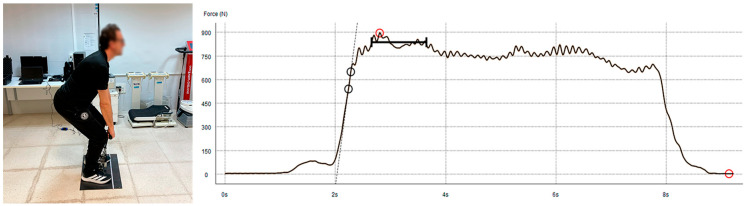
(**Left**): Deadlift position. (**Right**): Graph of the force generated during the exercise.

After the position was set, two instructions were provided. In the first one (pull a little), the participant had to pull progressively for two seconds, and in the second (to the maximum for five seconds), they had to do so to the best of their ability while maintaining the position. This was done to avoid an initial peak caused by an elastic movement that would distort the signal and the final recording (Figure 2). RFD was calculated as the slope of the force–time curve (ΔF/Δt) over the predefined early phase interval used in the dataset. For all inferential analyses, one observation per participant was retained (best trial, defined as the trial with the highest maximal force). Contraction onset was defined using the dynamometer/software-exported onset time marker (as provided in the dataset) rather than applying an additional force threshold algorithm; this criterion was used consistently for all participants to avoid subjective manual selection. The rate of force development (RFD) was calculated as the slope of the force–time curve within a fixed early phase window from the contraction onset (RFD = ΔF/Δt; e.g., 0–200 ms). The exported force–time signal used for RFD calculations was sampled at 160 Hz. No additional filtering or smoothing was applied in the present analyses beyond the processing performed by the device/software during acquisition and export (as provided in the dataset).

Two trials were performed for each participant. For inferential analyses, we retained one observation per participant, using the trial with the highest maximal force (best performance) [35]. For pain, we retained the maximum reported pain across both trials.

### 2.5. Statistical Analysis

We worked with a dataset evaluating strength in deadlift tasks among school-aged participants, including anthropometric and performance variables. The unit of analysis for inferential statistics was the participant (best trial). The main factors considered were sex (male/female) and educational stage (primary/secondary), with age (years) as a continuous variable. The performance variables included instantaneous force and peak/average force, as well as the rate of force development (RFD) (average and maximum values). Additionally, time stamps for action milestones (time_a, time_b) and indicators of execution-related pain were available for analysis. Within-session reliability between the two trials (A and B) was quantified for each performance variable using the intraclass correlation coefficient (ICC; two-way random-effects model, absolute agreement, single measurement ICC (A, 1)) and coefficient of variation (%CV). The typical error (TE) was computed as the SD of the intertrial differences divided by √2, and the %CV was calculated as the TE divided by the overall mean and multiplied by 100. The reliability estimates are reported in Table A1.

### 2.6. Preparation and Refinement

The data were inspected for missing values and for outliers. Records with implausible timestamps or force markers (time ≤ 0 or negative maximum force) were excluded. Categorical variables were recoded to readable and consistent labels (sex and stage), and the units were standardized.

### 2.7. Force–Time Curves to Characterize the Action Profile

The duration of each attempt was normalized by its actual length (t_norm = time/time_b), setting the peak to 1. Force was normalized by the individual peak (f_norm = force/maximumforce). Stylized curves were generated by interpolation between the operational anchors of the execution (start, time_a, and peak) and averaged for each group.

#### RFD Modeling and Age

To quantify the development of force production capacity with age, regression models were fitted with RFD as the dependent variable (average RFD and maximum RFD in separate analyses), including sex and stage as factors (main effects).

Linear, quadratic, and natural spline specifications (with three and four degrees of freedom) for age were evaluated by comparing the model fit using the AIC, BIC, and adjusted R^2^. Nested tests were used to assess the significant improvements in nonlinearity. Assumptions were checked by inspecting residuals and homoscedasticity, and estimates with robust standard errors were reported when necessary. The possible effect of pain on RFD was explored after adjusting for age, sex, and stage.

The models with splines (df = 4) showed a better fit than the linear ones, and the inclusion of the quadratic term significantly improved the fit compared to the linear model fit. Differences between sexes and stages were observed as shifts in the level, without strong interactions with age in the slope within the analyzed range. Pain indicators were not significantly associated with RFD after adjusting for the covariates.

### 2.8. Signal Processing and RFD Computation

For all inferential analyses, one observation per participant was retained (best trial, defined as the trial with the highest maximal force). Force onset was identified from the exported force–time trace as the start of the voluntary contraction using the system’s time markers, which were used consistently across all participants to avoid subjective manual selection. The rate of force development (RFD) was calculated as the slope of the force–time curve within a fixed time window from contraction onset, using the standard definition (RFD = DeltaF/Deltat).

Given the school-age population and to maximize robustness to small onset-detection variability, the primary RFD metric was computed over a 0–200 ms window from the onset (RFD_{0–200}). Peak RFD was defined as the maximum observed slope during the rising phase of the force–time curve derived from the exported data. The variables time_a and time_b correspond to the device-exported timing markers used for temporal referencing of the contraction and were used for curve alignment/normalization and quality control. Trials presenting implausible timing values or obvious recording artifacts (e.g., non-positive timing markers or non-physiological force patterns) were excluded prior to analysis.

Between-group effects of Sex and Educational Stage on scalar outcomes (e.g., maximal force and mean RFD) were tested using two-way between-subject models (Sex × Stage), followed by estimated marginal means with Bonferroni-adjusted post hoc comparisons.

All analyses were performed in R (R Core Team, v.4.5.2., Viena, Austria,) [36], mainly using dplyr (v.1.1.4.) and tidyr (v.1.3.2.) for data manipulation, ggplot2 (v.4.0.0.) [37] for visualization, and splines for nonlinear fitting with natural spline. The results were exported using openxlsx (v.4.2.8.1.) [38]. Reproducibility was ensured using the documented scripts and version control.

## 3. Results

### 3.1. Descriptive

The descriptive anthropometric data, by stage, sex, and strength development of the sample of Primary and Secondary students, were calculated using bidirectional comparisons (two-way between-subject models with factors Sex and Stage, with post hoc marginal comparisons and Bonferroni adjustment) and can be found in Table 1. The within-session reliability (ICC and %CV) for the main performance variables is reported in Table A1.

The distributions showed balanced sizes by stage and sex, with expected differences in the secondary education stage (*p* < 0.001).

### 3.2. Force–Time Curve Analysis (Raw and Time-Normalized Profiles)

The force–time curves were compared descriptively after time normalization. Group differences in summary outcomes derived from the force–time signal (e.g., maximal force and mean RFD) were assessed using two-way between-subject models with factors Sex and Stage, with post hoc marginal comparisons and Bonferroni adjustment (Table 2).

**Figure 3:**
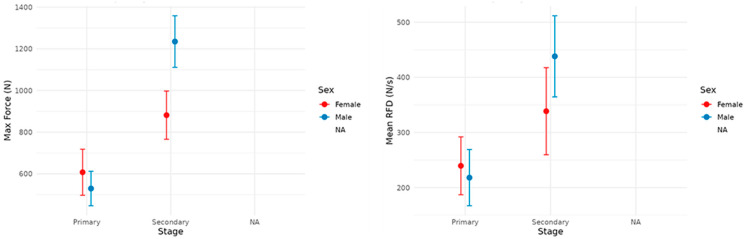
Maximum and average strength by sex for each educational stage.

Differences were found between Primary and Secondary in Men (*p* < 0.01) (Figure 3). In the Secondary trajectory, a greater proportion of relative strength was concentrated in the final phase of the action, and a more consistent relative peak was reached compared to the primary trajectory. In Women, the progression was more gradual in primary, and there was a greater relative accumulation toward the peak in secondary, mirroring the pattern observed in men. The average stage comparison confirmed that the Secondary displayed a relative trajectory with a larger area toward the end of the movement and a higher relative maximum, suggesting more efficient temporal coordination for reaching the peak.

### 3.3. Strength Normalized by Sex and Educational Stage

**Figure 4:**
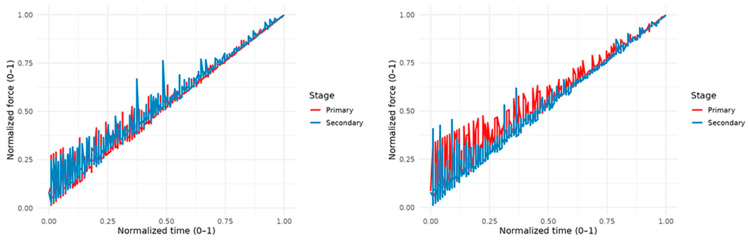
Normalization of strength by sex (women, (**left**); men, (**right**)) and educational stage.

By normalizing the actual duration (t_norm = time/time_b) and the individual peak force (f_norm), the age groups showed a progressive transition toward profiles with greater relative accumulation toward the peak as age increased, while maintaining the general shape of the action and isolating its magnitude (Figure 4).

The results by grade showed a shift in the shape toward a more consistent peak in higher grades, particularly in secondary grades. In Primary, the curves were more variable and less prominent toward the end. In the overall comparison of forms normalized by actual duration, Secondary showed a profile with greater relative density in the last third of the gesture than that of Primary. This reinforces that, beyond achieving higher peaks, the relative temporal distribution of effort is also reorganized beyond achieving higher peaks.

### 3.4. Differences Between Sex and Stage

Table 2 shows the post hoc marginal comparisons (emmeans) with Bonferroni adjustments. Effect sizes were generally small and should be interpreted cautiously because several confidence intervals were wide and overlapped with zero.

There were no sex differences in primary education in either the maximal strength or mean RFD (computed as RFD_{0–200} from contraction onset; see Section 2) (*p* > 0.65). In Secondary education, the sex difference in maximal strength approached significance (*p* = 0.055), with higher values in boys; no differences were observed in the mean RFD (*p* = 0.124). By educational stage, boys showed greater maximal strength in Secondary than in Primary (*p* = 0.0017), whereas in girls, this difference was not significant (*p* = 0.275). The effects of stage on RFD were not significant in girls (*p* = 0.989) but were at the threshold in boys (*p* = 0.051). Effect sizes (Cohen’s d) were small in most comparisons, with a small to moderate effect favoring boys in secondary school for maximal strength measurements.

### 3.5. Impact of Perceived Pain

In exploratory analyses adjusted for age, sex, and stage, the indicator of pain during execution did not show consistent associations with RFD metrics. These findings suggest that pain did not significantly alter the relative form of action or the aggregated metrics in this dataset.

## 4. Discussion

Strength is a fundamental ability that should be developed early in life [39]. It is an excellent indicator of the level of muscular fitness on which functional variables, proprioceptive abilities, and various health markers depend [40,41]. Conditioning at an early age leads to evolutionary changes that, if not addressed, result in negative adaptations that must be counteracted in adulthood, such as a pediatric back that has not been consistently conditioned and compensated [42]. Maintaining this ability is essential to ensure incident-free practice, regardless of an individual’s conditioning frequency or goals [43].

This study helps map the traceability between the educational and adult stages, providing a comprehensive perspective that is supported by other studies [44,45] and the expected development of strength at an early age. At this point, special attention should be given to the trunk, as it allows the transfer of strength between the upper and lower limbs. This mechanism is replicated in practically all functional movements and requires a good response from the paravertebral musculature [46], which is the result of the interaction between the skeletal, muscular, and neural subsystems, ensuring efficient strength development when muscle coactivation occurs.

The calculation of RFD is an accurate measurement that allows one to indicate the strength profile and its stability, making it possible to anticipate and provide advice on the types of practices that would be suitable within Physical Education or a regular exercise program [47,48]. On the other hand, it can identify appropriate patterns of intra- and intermuscular coordination, which greatly contribute to proper strength development, benefiting not only the practice profile but also reducing the risk of muscle injury [49].

The RFD profile of the study revealed that it would be highly beneficial to work on strength elements involving speed to improve the recruitment of motor units, and above all, to do so consistently over time with each attempt to provoke better adaptations and greater possibilities of techniques when generating force. In line with other studies [50], strength increased, especially in boys, between the two stages, showing a steady improvement that was not as pronounced in girls, reaffirming the quintessential anabolic nature that affects males during this period of their lives.

Safety in load mobilization is crucial, especially at these ages, and the study has shown that the deadlift has a low incidence of pain because it does not negatively affect force production. Therefore, it can be stated that this is an appropriate, accessible, and low-risk exercise. Its global nature and easy assimilation help to express strength fully, and it stands out as the option to choose over other, more complex alternatives for young people, such as the squat [25] before exercises like the hip thrust, as it allows better observation of changes in strength deficits [51]. The specific bibliography supports the deadlift exercise for its preventive effects, especially concerning the lumbar region [52,53], and as a benchmark for indicating the voluntary strength of the trunk muscles. Furthermore, since there is extensive specific literature available for adults, it allows for comparisons between age groups to monitor which aspects should be improved, in both the fitness field and performance-oriented settings [54].

The practical and fundamental pedagogical implications that can be drawn are rich, such as the need to work on the different manifestations of strength with an explosive character, promoting efficient movement chains that can be transferred to various forms of exercise, such as jumps, accelerations, and complex coordinations. In another aspect, the promotion of using accessible and objective protocols and measurement tools in the school environment [55,56] for carrying out mass assessments that may have a more generalized nature and provide immediate feedback [31], similar to the mobile applications already embraced by young people.

Despite the findings of this study, there are some limitations to consider. Post-test studies should be conducted to observe neuromuscular plasticity and the primary prevention of sarcopenia or future functional limitations. Additionally, conducting the test with exercise and dynamic protocols could establish a comparison with isometric work in the future.

Comparing more global-type exercises or checking the change in the strength profile after the induction of fatigue could also be viable elements of a study that might provide answers to some of the causes of muscle-joint injuries of the trunk. In the case of the measurements taken, the definition of the onset of pull should be indicated, as this will greatly affect the RFD results. In addition, we quantified the within-session reliability and measurement error between the two trials (ICC, typical error, and %CV; see Table A1); however, future studies should also examine the between-session test–retest reliability using separate testing days and a standardized algorithmic definition of contraction onset, particularly for early phase RFD outcomes.

## 5. Conclusions

These results suggest that the isometric deadlift is feasible in school settings and that force–time characteristics differ by educational stage, particularly for maximal force in boys’ performance. However, the present cross-sectional data do not allow for conclusions about injury prevention or long-term clinical benefits. The greatest improvement and difference occurred in Secondary Education students, in line with the evolutionary logic of adolescence. The isometric deadlift exercise is a technically accessible alternative for calculating maximum strength and presents low levels of pain that do not interfere with its performance. Future studies should compare dynamic alternatives and interventions in exercise programs.

## Figures and Tables

**Table 1:** Descriptive data of the sample.

Stage	Sex	n	Age	Weight (kg, SD)	Height (Cm, SD)	MaxForce (N, SD)	MeanRFD (N·s^−1^, SD)	PeakRFD (N·s^−1^, SD)
Primary	Female	49	10.24 ± 0.90	40.6 ± 9.412	145.17 ± 8.43	607.65 ± 395.11	239.51 ± 187.60	2017.80 ± 2327.34
	Male	47	10.17 ± 0.92	41.29 ± 10.18	143.37 ± 7.78	529.61 ± 292.55	218.09 ± 108.45	2305.43 ± 2186.15
Secondary	Female	51	13.73 ± 1.04	53.95 ± 10.03	159.55 ± 7.05	881.61 ± 425.43	338.58 ± 290.64	2449.07 ± 1950.84
	Male	80	13.56 ± 1.07	58.55 ± 13.31	166.51 ± 9.69	1234.80 ± 564.75	438.12 ± 335.77	3574.11 ± 2777.68

Note: Kg = Kilograms; SD: Standard deviation; Cm: Centimeters; N·= Newtons; RFD (Mean RFD and Peak RFD) is expressed as N·s^−1^ (N/s).

**Table 2:** Strength comparisons by sex and stage.

Outcome	Contrast	Context	Estimate	SE	*p*_adj	d	d_low	d_high
Max Force	Female–Male	Primary	1.881	22.579	0.934	0.031	−0.369	0.431
		Secondary	−38.438	19.937	0.055	−0.283	−0.637	0.073
Mean RFD		Primary	59.140	142.139	0.678	0.127	−0.274	0.527
		Secondary	−193.725	125.506	0.124	−0.235	−0.589	0.120
Max Force	Primary–Secondary	Female	−24.322	22.231	0.275	−0.364	−0.761	0.034
		Male	−64.641	20.325	0.002	−0.478	−0.842	−0.112
Mean RFD		Female	2.013	139.945	0.989	0.004	−0.390	0.398
		Male	−250.852	127.947	0.051	−0.302	−0.664	0.060

Note: SE = Standard error; *p*_adj. = *p*-value adjusted for multiple comparisons (Bonferroni); d = Cohen’s d, standardized effect size; d_low/d_high = lower and upper limits of the confidence interval.

## Data Availability

The datasets used and analyzed in the current study are available from the corresponding author upon reasonable request, owing to privacy and ethical restrictions.

## Abbreviations

The following abbreviations are used in this manuscript.

RFD	Rate of force development

## Appendix A

**Table A1:** Within-session reliability (two trials A and B) of the main performance variables.

Variable	Trial1	Trial2	n	ICC A1	CV Percent	Typical Error
Max force N	Force A	Force B	227	0.073	71.4	339.86
RFD N per s	RFD A	RFD B	226	0.139	292.5	602.85
Time to peak ms	Time A	Time B	227	0.099	31.1	1037.07

Note: ICC values correspond to the intraclass correlation coefficient computed using a two-way random-effects model with absolute agreement for a single measurement (ICC(A,1)). The typical error (TE) was calculated as the SD of the intertrial differences divided by √2. The coefficient of variation was computed as %CV = (TE/overall mean) × 100. A higher ICC indicates better relative reliability, whereas a lower %CV indicates a lower measurement error.
